# Insights Into the Antiviral Pathways of the Silkworm *Bombyx mori*

**DOI:** 10.3389/fimmu.2021.639092

**Published:** 2021-02-11

**Authors:** Liang Jiang

**Affiliations:** ^1^State Key Laboratory of Silkworm Genome Biology, Southwest University, Chongqing, China; ^2^Biological Science Research Center, Southwest University, Chongqing, China

**Keywords:** immunity, signaling pathway, virus, antivirus, insect, silkworm

## Abstract

The lepidopteran model silkworm, *Bombyx mori*, is an important economic insect. Viruses cause serious economic losses in sericulture; thus, the economic importance of these viruses heightens the need to understand the antiviral pathways of silkworm to develop antiviral strategies. Insect innate immunity pathways play a critical role in the outcome of infection. The RNA interference (RNAi), NF-kB-mediated, immune deficiency (Imd), and stimulator of interferon gene (STING) pathways, and Janus kinase/signal transducer and activator of transcription (JAK/STAT) pathway are the major antiviral defense mechanisms, and these have been shown to play important roles in the antiviral immunity of silkworms. In contrast, viruses can modulate the prophenol oxidase (PPO), phosphatidylinositol 3-kinase (PI3K)/protein kinase B (Akt), and the extracellular signal-regulated kinase (ERK) signaling pathways of the host to elevate their proliferation in silkworms. In this review, we present an overview of the current understanding of the main immune pathways in response to viruses and the signaling pathways modulated by viruses in silkworms. Elucidation of these pathways involved in the antiviral mechanism of silkworms furnishes a theoretical basis for the enhancement of virus resistance in economic insects, such as upregulating antiviral immune pathways through transgenic overexpression, RNAi of virus genes, and targeting these virus-modulated pathways by gene editing or inhibitors.

## Introduction

Virus infection poses a serious threat to human health and agricultural production. As the only fully domesticated insect, the lepidopteran model silkworm, *Bombyx mori*, is economically important for silk production. Sericulture is one of the main sources of income for farmers in many developing countries (1, 2). However, viral diseases have caused losses of nearly 16% of the potential cocoon production each year in sericulture, which are induced mainly by the *Bombyx mori nucleopolyhedrovirus* (BmNPV), *Bombyx mori cytoplasmic polyhedrosis virus* (BmCPV), or the *Bombyx mori bidensovirus* (BmBDV) (1).

Insects mainly rely on innate immunity to defend against invading pathogens, and immune pathways play an important role in this process. Although some host signaling pathways can be modulated by viruses to elevate virus proliferation, targeting these pathways can also inhibit virus infection. In this review, we present an overview of the main pathways involved in the antiviral mechanism of silkworms. Such knowledge could provide a theoretical basis for strategies for control of viral diseases in economic insects.

## Characteristics of Silkworm Viruses

Among the three major pathogenic viruses of silkworms, the BmNPV, a member of the Baculoviridae family having a circular double-stranded DNA genome (3), is the most prevalent threat to sericulture in almost all countries (1). The viral DNA combines with capsid proteins to form a nucleocapsid that is contained within an envelope (1, 3). The BmNPV replication cycle has two virion phenotypes: (1) the occlusion-derived virus that is transmitted among hosts, and packaged and protected in an occlusion body (4, 5), and (2) the budded virus that spreads throughout the host. The BmCPV belongs to the *Cypovirus* genus of the *Reoviridae* family, and its genome consists of ten discrete double-stranded RNA (dsRNA) segments (6, 7). The BmCPV particles contain nucleic acid and protein capsid, and they are non-enveloped and occluded within polyhedral bodies (6, 7). The BmBDV belongs to the *Bidensovirus* genus of *Bidnaviridae* family, and has two geographical variants, BmDNV-2 and BmDNV-Z (8–10). The BmBDV virions are non-enveloped and assembled by a protein capsid and nucleic acid, with their viral genome consisting of two linear non-homologous single-stranded DNA segments (8–10).

These viruses invade the silkworm larvae mainly via oral infection. The BmNPV can infect almost all tissues of the silkworm whereas the BmCPV and BmBDV can only infect the silkworm midgut (1). Some silkworm strains are resistant to the viruses at any viral dose (1, 9). For example, the *nsd-2* mutation is caused by a 6-kb deletion in the open reading frame of +^*nsd*−2^ and imparts resistance to the BmDNV-2 (9). However, the receptor and major resistance genes to the BmNPV and BmCPV have not been identified in silkworm. The BmN and BmE are two cell lines commonly used in silkworm research, which are derived from the ovary and embryonic cells of silkworm, respectively. The BmNPV can infect the two cell lines, unlike the BmCPV and BmBDV; therefore, most silkworm antiviral research is focused on the BmNPV (11–17), a few on the BmCPV (18, 19), and very few on the BmBDV (20).

## Silkworm Antiviral Immune Pathways

The antiviral defense mechanism of silkworms mainly relies on innate immunity, including the RNA interference (RNAi), NF-kB-mediated pathways, and Janus kinase/signal transducer and activator of transcription (JAK/STAT) pathway (19, 21–24). Among these immune responses, RNAi is the major defense strategy against viruses in insects (23, 25).

### RNAi Pathways

**Figure 1:**
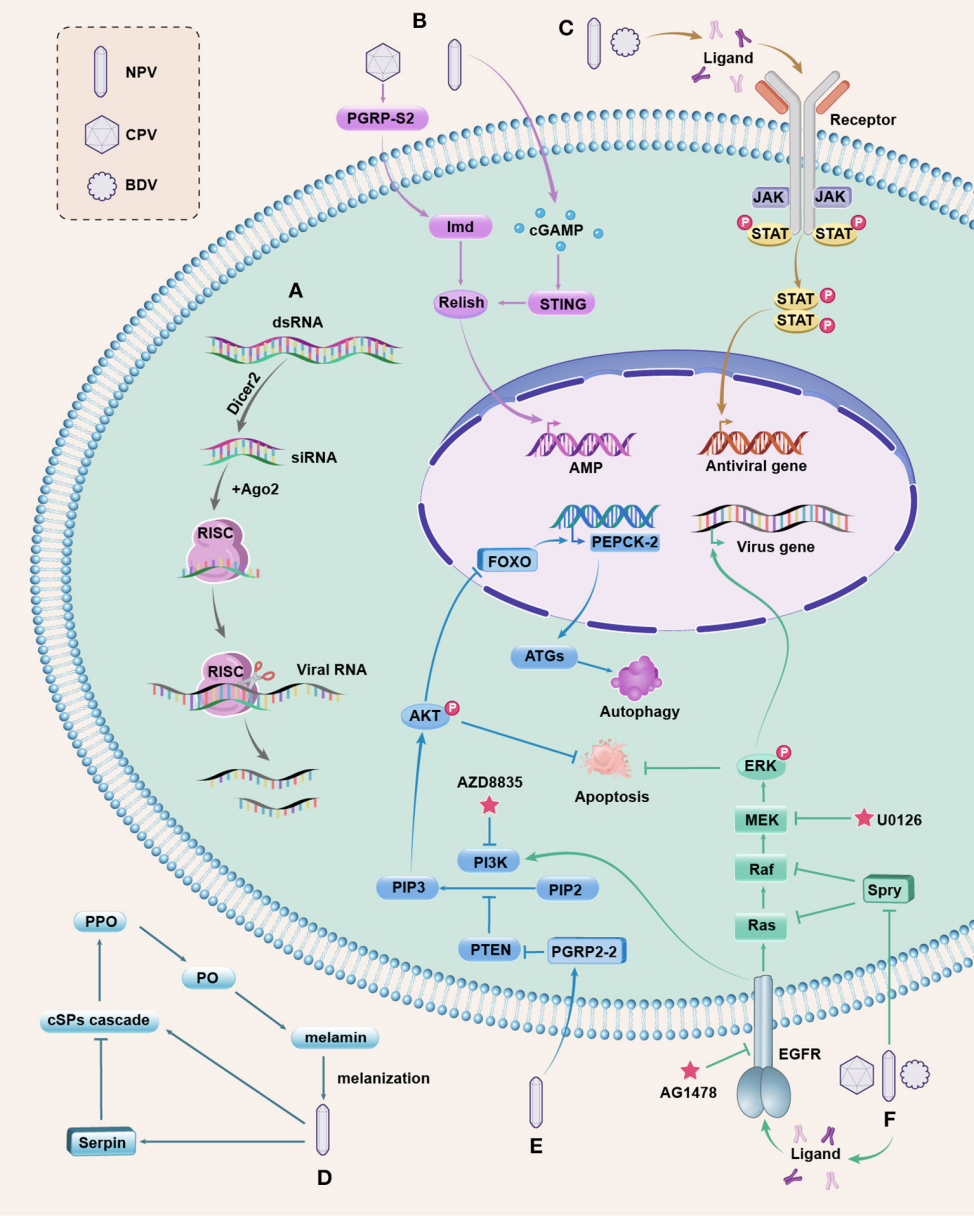
Antiviral pathways in silkworm. **(A)** The siRNAi pathway is activated by viral dsRNA, which is cleaved into siRNAs by Dicer2. Ago2 is associated with one strand of siRNA to form RISC that can target and cleave the viral RNA to inhibit viral replication. **(B)** The NF-kB-mediated, Imd, and STING pathways. BmCPV induces the extracellular BmPGRP-S2 to active Imd and the downstream NF-kB ortholog Relish; BmNPV infection triggers the production of cGAMP to activate BmSTING for processing Relish. Activated Relish is translocated to the nucleus to initiate the transcription of AMP. Whether AMPs have antiviral function in silkworms needs further study. **(C)** The JAK/STAT pathway. The extracellular ligands bind to JAK associated receptors upon stimulation, leading to the activation of JAKs, and then cytosolic STATs are phosphorylated, forming the STAT dimers, which are translocated to the nucleus to regulate the expression of antiviral genes. **(D)** The PPO pathway is initiated by recognizing invading microbes, and then the extracellular cSP cascade is activated to convert the zymogen PPO to active PO. PO catalyzes the formation of melanin, resulting in melanization that kill the microbes. This pathway is negatively regulated by serpins, and baculovirus can induce serpins to suppress the melanization response of host insects for survival. **(E)** The PI3K/Akt pathway. Activated PI3K converts PIP2 into PIP3 to cause Akt phosphorylation (p-Akt). PTEN is a negative regulator of the PI3K/AKT pathway. BmNPV induces BmPGRP2-2 to suppress PTEN, resulting in increased p-Akt that inhibits cell apoptosis. Upregulated p-Akt also causes the inhibitory phosphorylation of the transcription factor FOXO, decreasing the expression of *BmPEPCK-2* and resulting in reduced autophagy genes (ATGs) expression, thereby blocking host autophagy. The inhibited apoptosis and autophagy are beneficial for viral replication. The PI3K inhibitor AZD8835 can decrease the mortality of silkworms infected with BmNPV. **(F)** The ERK pathway. Upon viral infection, the extracellular ligands activate EGFR (a receptor tyrosine kinase) to promote ERK phosphorylation (p-ERK) through the activation of Ras to the Raf/MEK/ERK phosphorylation cascade. p-ERK can regulate the transcription of viral genes and inhibit apoptosis. The Spry protein is a negative regulator of EGFR/ERK pathway that inhibits Ras or Raf, and both DNA and RNA viruses can downregulate Spry to increase p-ERK to ensure viral reproduction. AG1478 is a specific inhibitor of EGFR and U0126 binds to MEK to prevent p-ERK. The EGER also participates in the activation of PI3K by BmNPV. These pathways are integrated and are responsive to one another, which are complex and merit further investigation.

There are three RNAi-related pathways in insects, including the small interfering RNA (siRNA) pathway, microRNA (miRNA) pathway, and the PIWI-associated RNA (piRNA) pathway (26). When challenged with viruses, the siRNA pathway is activated by the dsRNA that is commonly generated as a byproduct of viral replication (27, 28). The Dicer2 enzyme recognizes viral dsRNA and processes the dsRNA into siRNAs. One strand of duplex siRNA is associated with Ago2 to form the RNA-induced silencing complex (RISC), and then directs RISC to the viral RNA target through base pairing. Subsequently, Ago2 cleaves the viral RNA, inhibiting viral replication (25, 27, 28) (Figure 1A). The expressions of both Ago2 and Dicer2 were not induced by silkworm viruses (21). However, the results of deep sequencing revealed that a large number of viral siNRA (~20 nucleotides) was generated in insect hosts infected with baculovirus (29) and BmCPV (30), indicating that the RNAi response is an important antiviral defense of hosts. Overexpression of Ago2 and Dicer2 can improve the susceptibility of silkworm to dsRNA (31). Expression of dsRNA targeting the viral genes of BmNPV (13), BmCPV (18), and BmBDV (20) in transgenic silkworms substantially decreased the viral mRNA content and silkworm mortality after viral infection. The siRNA pathway is the predominant mechanism responsible for antiviral activity in insects (27, 28). For the applications and challenges of insect RNAi, please refer to the recent reviews (32, 33).

The miRNAs are small noncoding RNAs that can bind to target genes and regulate their expression (34). The miRNA pathway is involved in the interaction between silkworm and viruses (23, 35). Virus-encoded miRNA can facilitate viral multiplication. For example, BmNPV-miR-1 (35) and BmNPV-miR-3 (36) can enhance BmNPV infection via regulating the exportin-5 cofactor Ran and the viral *P6.9* expression, respectively; BmCPV-miR-1 could facilitate target gene *BmIAP* expression and BmCPV replication (37). Similarly, silkworm-encoded miRNA could be regulated to promote viral proliferation. For example, bmo-miR-274-3p, whose inhibition enhances target viral *NS5* expression and facilitates BmCPV replication, was downregulated in a BmCPV-infected silkworm midgut (38). Additionally, host miRNA can inhibit viral proliferation. For example, bmo-miR-2819 can downregulate the *ie-1* gene of BmNPV to suppress viral multiplication (39); although bmo-miR-278-3p could decrease target gene *IBP2* expression and increase BmCPV mRNA, it is downregulated and *IBP2* is upregulated in BmCPV-infected silkworms (40). The contribution of the miRNA pathway is minor in the RNAi antiviral defense of insects. In contrast to siRNAs and miRNAs, piRNAs are derived from single stranded RNA precursors (23). The role of the piRNA pathway in the antiviral response of insect models has been reviewed recently (41), however, of which the exact roles in the interaction between silkworm and its major pathogenic viruses are unclear, having few relevant reports so far (42, 43).

### NF-kB-Mediated Antiviral Pathways

The Imd and Toll pathways are canonical NF-kB-dependent pathways involved in the innate immunity of insects, wherein they activate the downstream antimicrobial peptide (AMP) genes transcription mediated by two distinct orthologs of the NF-kB transcription factor (19, 25, 44). The NF-kB ortholog Relish is the terminal transcription factor for the Imd pathway, whereas the Dorsal and Dorsal-related immune factor (Dif) function in the Toll pathway (25). Toll pathway responds to Gram-positive bacteria and fungi infections, whereas Imd pathway responds Gram-negative bacteria (19, 25). The transmembrane receptors peptidoglycan recognition protein (PGRP)-LC and the intracellular PGRP-LE sense the diaminopimelic acid-type peptidoglycan of Gram-negative bacteria, and transmit the signal to the adaptor molecule Imd, which is essential for the activation of Relish (25, 45). The Imd and Toll pathways have been shown to play a role in the antiviral immunity of *Drosophila* (25, 46–48). AMPs seems to have antiviral function in *Drosophila*, but their exact antiviral mechanisms are still unknown and more in-depth researches are needed (49).

Our research showed that *BmPGRP-S2* was induced by BmCPV in the silkworm midgut (7). Further experiments revealed that BmPGRP-S2 was a secreted protein, which may recognize a certain viral component and then transmit the signal to downstream molecules, and its overexpression increased the expression of *BmImd, BmRelish*, and *AMPs* and decreased silkworm mortality after BmCPV infection (19) (Figure 1B). These results indicate that the Imd pathway is involved in the defense against the RNA virus in silkworms. However, the function of this pathway in DNA virus-infected silkworms is not yet known. There have been few reports on the Toll pathway involved in antiviral immunity in silkworms. Recently, the stimulator of interferon genes (STING) has been reported to provide antiviral immunity against BmNPV in silkworms by promoting NF-kB activation (22). Production of cyclic guanosine monophosphate–adenosine monophosphate (cGAMP) is triggered upon BmNPV infection, inducing the BmSTING activation to process BmRelish, and then the activated BmRelish is translocated to the nucleus to initiate the transcription of AMP (22) (Figure 1B). The aforementioned result revealed that the NF-kB-mediated, Imd, and STING pathways play important roles in silkworm antiviral defense, but the antiviral mechanisms of the two pathways are only partially elucidated and need more experimentation. Deciphering the roles of Toll pathway in silkworm antiviral immunity remains a challenging task.

### JAK/STAT Pathway

JAK/STAT signaling is an important pathway involved in multiple cellular processes such as cell proliferation and immune regulation in insects (21, 25). This pathway contains a diverse family of extracellular ligands such as cytokine and growth factors, transmembrane receptors, JAK tyrosine kinases that are associated with the intracellular part of the receptor, and STAT proteins (25, 50). Following stimulation, a ligand binds to the extracellular part of the JAK-associated receptors, leading to the activation of JAKs. Subsequently, cytosolic STATs are recruited to the JAK/receptor complex, and then phosphorylated, forming the STAT dimers, which are translocated into the nucleus and bound to the DNA promoters of the target genes to regulate their expression (25, 50) (Figure 1C).

The insect JAK/STAT pathway activation mechanism has been well-established in *Drosophila* and mosquito (25, 51–53). There has been growing evidence that the JAK/STAT pathway may be functionally analogous to the mammalian interferon system (51). The JAK/STAT pathway has been shown to respond to viral infections in *Drosophila* by regulating the production of downstream effector molecules, including the AMPs (25, 53). The BmNPV and BmBDV, unlike the BmCPV, induce the expression of *BmSTAT* in silkworms, implying that the JAK/STAT pathway could be activated by the DNA viruses in silkworms (21). Overexpression of *BmSTAT* in BmN cells increased the number of cells in the G2 phase of the cell cycle (54) and host resistance to BmNPV, but not to BmCPV (55). Additionally, inhibition of Hsp90 can cause upregulation of *BmSTAT* expression and suppression of BmNPV replication in the BmN cell (56), but it is not clear how Hsp90 can be linked to JAK/STAT. The extracellular ligand and effector molecules of this pathway in response to viral infection in silkworms have not been clearly identified and merit further investigation.

## Virus-Modulated Host Signaling Pathways

During the interaction between the insects and viruses, several host signaling pathways including the prophenol oxidase (PPO), phosphatidylinositol 3-kinase (PI3K)/protein kinase B (Akt), and the extracellular signal-regulated kinase (ERK) pathways have been reported to be modulated by viruses to elevate viral proliferation. For example, baculovirus induces *Bmserpin2* to inhibit the melanization reaction mediated by the PPO pathway, which also induces *BmPGRP2-2* to suppress *PTEN*, resulting in increased p-Akt that can inhibit cell apoptosis and autophagy. Meanwhile, silkworm viruses usurp the ERK pathway by downregulating *BmSpry* (57–60). It is noteworthy that targeting these hijacked host pathways can inhibit viral proliferation in silkworm.

### PPO Pathway

Melanization reaction, mediated by the PPO pathway, is an important immune response in insect plasma and plays an essential role in the wound healing and killing of microbes (61, 62). This process is initiated by the recognition of invading microbes, and then the extracellular clip-domain serine protease (cSP) cascade is activated to convert the zymogen PPO to active phenoloxidase (PO). PO catalyzes the oxidation of phenols to form quinones and melanin, wherein the rapid polymerization of melanin at infection sites can kill and immobilize microbes (61–63) (Figure 1D). The melanization can kill baculovirus *in vitro* (64, 65). However, the PPO pathway is negatively regulated by serpins, and baculovirus can induce serpins to suppress the melanization response of host insects for survival (57, 64). *Bmserpin2* was upregulated in silkworms after BmNPV infection. Furthermore, knockdown of *Bmserpin2* can increase PO activity and decrease viral multiplication (57). The mechanism by which melanization contributes to the killing of pathogens remains elusive.

### PI3K/Akt Pathway

The PI3K /Akt pathway plays an important role in regulating a number of cellular processes (66–68). Activation of PI3K can occur through the binding of a variety of ligands, including several growth factors to the receptor tyrosine kinases (RTKs). Activated PI3K then converts the substrate phosphatidylinositol 4, 5-bisphosphate (PIP2) into phosphatidylinositol (3,4,5)-trisphosphate (PIP3), and PIP3 causes the phosphorylation of Akt (p-Akt). Akt is considered a central mediator of the PI3K pathway. Active Akt drives cell proliferation, survival, apoptosis, and metabolism through the inhibitory phosphorylation of several substrates, including related kinases, signaling proteins, and the transcription factor forkhead box O (FOXO) (66, 69–71). BmFOXO directly upregulates *BmPEPCK-2*, and overexpression of *BmFOXO* and *BmPEPCK-2* can increase the expression of autophagy genes *ATG6/7/8* (17, 72). In addition, phosphatase and tensin homolog (PTEN) protein causes the dephosphorylation of PIP3, resulting in the suppression of the PI3K/AKT pathway (73).

A number of studies have demonstrated that many viruses can activate the PI3K/AKT pathway for their efficient proliferation (58, 66, 74, 75). The BmNPV induces the peptidoglycan recognition protein BmPGRP2-2 to suppress *PTEN*, resulting in increased p-Akt that can inhibit cell apoptosis (58). Meanwhile, the upregulation of p-Akt attenuates the activity of FOXO and decreases the expression of *BmPEPCK-2* and *ATG6/7/8*, thereby blocking host autophagy (17, 58, 72) (Figure 1E). The inhibited apoptosis and autophagy are beneficial for viral replication. However, which viral components are recognized by BmPGRP2-2 is unclear and needs further study. The PI3K/AKT pathway is a target for the treatment of many diseases (68, 70). The PI3K inhibitor AZD8835 can decrease the mortality of silkworms infected with BmNPV by blocking the p-Akt and suppressing viral proliferation (76), implying a promising antiviral strategy for silkworms.

### ERK Pathway

ERKs are serine/threonine kinases activated by a variety of extracellular stimuli such as growth factors, environmental stresses, and microbial infections, and can transduce downstream cellular responses, including cell differentiation, survival, and apoptosis (77–80). Activation of the ERK pathway is required for efficient infection by many viruses (59, 80). One major class of ERK regulators is the RTK family. Upon stimulation, the extracellular ligands activate RTKs to promote the phosphorylation of ERK (p-ERK) by the activation of the small GTPase Ras to the Raf (MAP3K)/MEK (MAP2K)/ERK (MAPK) phosphorylation cascade. The ERKs then control transcription by phosphorylating various transcription factors in the nucleus or control targets in the cytoplasm (77, 78, 81, 82).

The epidermal growth factor receptor (EGFR) belongs to the RTK family (78, 81). The BmEGFR plays an important role in BmNPV infection, which participates in the activation of ERK and PI3K/Akt pathways by the virus. Moreover, activated ERK regulates the transcription of late viral genes and inhibits apoptosis (83). Additionally, Spry is a negative regulator of the EGFR/ERK pathway through the inhibition of Ras or Raf, and the overexpression of *BmSpry* suppressed p-ERK and BmNPV replication in BmE cells (84) (Figure 1F). Further research has found that *BmSpry* was decreased and p-ERK was increased in silkworms after infection with BmNPV, BmCPV, or BmBDV, and the knockdown of *BmSpry* in transgenic silkworms caused increased p-ERK, viral content, and mortality after infection with the three viruses, revealing that both DNA and RNA viruses usurp the ERK pathway to ensure viral reproduction (60). AG1478 is a specific inhibitor of EGFR tyrosine kinase activity (85) and the inhibitor U0126 binds to MEK to prevent p-ERK (86). The two inhibitors can inhibit p-ERK and BmNPV in BmE cells (83), but the inhibitory effect in silkworm larvae needs further test. The ERK pathway plays important roles in regulating the outcome of viral infection in silkworms, and the mechanisms remain to be fully elucidated.

## Conclusions and Future Prospects

Antiviral mechanisms are a worldwide problem and research hotspot. Insect-virus interactions may provide information on a vast repertoire of antiviral immune mechanisms (27). Results from the silkworm-virus model clearly show that there are multiple layers of antiviral defense that rely on conserved but also divergent pathways. For example, RNAi is a conserved antiviral mechanism among different insects, and it is the major antiviral response against both DNA and RNA viruses in silkworms. Meanwhile, NF-kB-mediated pathways are involved in antiviral immunity in silkworms but divergent responses to different viruses, such as BmCPV induces *BmPGRP-S2* and *Imd* to activate Relish whereas BmNPV activates cGAMP and STING to process Relish. Additionally, RNAi inhibits viral replication by cleaving the viral RNA while NF-kB-dependent antiviral immunity may based on AMPs. The multi-level response is beneficial to antiviral defense of host.

It is now apparent that these antiviral pathways are integrated and are responsive to one another, providing a pathogen-specific response. For example, the ERK and PI3K/Akt pathways have all been reported to interact with the JAK/STAT pathway (25), and the melanization and Toll pathways have also been found to interact (63). However, the integrated mechanisms of these pathways are complex, that is, the mechanisms by which baculovirus activate the ERK and PI3K/Akt pathways through EGFR may be different (83) and merit further investigation. Meanwhile, some mechanisms are tissue-specific or virus-specific, highlighting the importance of the investigation of virus–host interactions in the right context.

Coevolution between hosts and viruses favors the development of immune evasion mechanisms through modulation of the host signaling pathways by the pathogen (87). Targeting these hijacked pathways using inhibitors and knocking out their key regulators via gene editing would be a promising strategy to improve silkworm resistance. Meanwhile, RNAi of viral genes and overexpression of antiviral genes can enhance antiviral capacity of transgenic silkworms (1). Additionally, upregulation of antiviral immune pathways in transgenic silkworms is an available antiviral strategy. For the enhancement of host antiviral capacity and major issues in silkworm antiviral studies, please refer to our other review (87). These studies on antiviral pathways would be very instructive as they would reveal original antiviral strategies for the protection of beneficial insects and the target pathways hijacked by viruses for pest control.

## Author Contributions

LJ: drew figure, wrote the article, and supervision.

## Conflict of Interest

The author declares that the research was conducted in the absence of any commercial or financial relationships that could be construed as a potential conflict of interest.

## References

[1] Jiang L Xia QY. The progress and future of enhancing antiviral capacity by transgenic technology in the silkworm *Bombyx mori*. Insect Biochem Molec. (2014) 48:1–7. 10.1016/j.ibmb.2014.02.003 24561307

[2] Goldsmith MR Shimada T Abe H. The genetics and genomics of the silkworm, *Bombyx mori*. Annu Rev Entomol. (2005) 50:71–100. 10.1146/annurev.ento.50.071803.130456 15355234

[3] Gomi S Majima K Maeda S. Sequence analysis of the genome of *Bombyx mori* nucleopolyhedrovirus. J Gen Virol. (1999) 80:1323–37. 10.1099/0022-1317-80-5-1323 10355780

[4] Blissard GW Theilmann DA. Baculovirus entry and egress from insect cells. Annu Rev Virol. (2018) 5:113–39. 10.1146/annurev-virology-092917-043356 30004832

[5] Rahman MM Gopinathan KP. Systemic and *in vitro* infection process of *Bombyx mori* nucleopolyhedrovirus. Virus Res. (2004) 101:109–18. 10.1016/j.virusres.2003.12.027 15041178

[6] Cao GL Meng XK Xue RY Zhu YX Zhang XR Pan ZH. Characterization of the complete genome segments from BmCPV-SZ, a novel *Bombyx mori* cypovirus 1 isolate. Can J Microbiol. (2012) 58:872–83. 10.1139/w2012-064 22712678

[7] Jiang L Peng ZW Guo YB Cheng TC Guo HZ Sun Q. Transcriptome analysis of interactions between silkworm and cytoplasmic polyhedrosis virus. Sci Rep-Uk. (2016) 6. 10.1038/srep24894 27118345 PMC4847007

[8] Wang YJ Yao Q Chen KP Wang Y Lu J Han X. Characterization of the genome structure of *Bombyx mori* densovirus (China isolate). Virus Genes. (2007) 35:103–8. 10.1007/s11262-006-0034-3 17048112

[9] Ito K Kidokoro K Sezutsu H Nohata J Yamamoto K Kobayashi I. Deletion of a gene encoding an amino acid transporter in the midgut membrane causes resistance to a Bombyx parvo-like virus. Proc Natl Acad Sci USA. (2008) 105:7523–7. 10.1073/pnas.0711841105 18495929 PMC2391135

[10] Sun Q Guo HZ Xia QY Jiang L Zhao P. Transcriptome analysis of the immune response of silkworm at the early stage of *Bombyx mori* bidensovirus infection. Dev Comp Immunol. (2020) 106:601. 10.1016/j.dci.2019.103601 31899306

[11] Jiang L Wang GH Cheng TC Yang Q Jin SK Lu G. Resistance to *Bombyx mori* nucleopolyhedrovirus via overexpression of an endogenous antiviral gene in transgenic silkworms. Arch Virol. (2012) 157:1323–8. 10.1007/s00705-012-1309-8 22527866

[12] Jiang L Cheng TC Zhao P Yang Q Wang GH Jin SK. Resistance to BmNPV via overexpression of an exogenous gene controlled by an inducible promoter and enhancer in transgenic silkworm, *Bombyx mori*. PLoS ONE. (2012) 7:e41838. 10.1371/journal.pone.0041838 22870254 PMC3411602

[13] Jiang L Zhao P Wang GH Cheng TC Yang Q Jin SK. Comparison of factors that may affect the inhibitory efficacy of transgenic RNAi targeting of baculoviral genes in silkworm, *Bombyx mori*. Antivir Res. (2013) 97:255–63. 10.1016/j.antiviral.2012.12.020 23274787

[14] Jiang L Zhao P Cheng TC Sun Q Peng ZW Dang YH. A transgenic animal with antiviral properties that might inhibit multiple stages of infection. Antivir Res. (2013) 98:171–3. 10.1016/j.antiviral.2013.02.015 23466668

[15] Chen SQ Hou CX Bi HL Wang YQ Xu J Li MW. Transgenic clustered regularly interspaced short palindromic repeat/Cas9-mediated viral gene targeting for antiviral therapy of *Bombyx mori* nucleopolyhedrovirus. J Virol. (2017) 91:16. 10.1128/JVI.02465-16 28122981 PMC5375672

[16] Jiang L Xie E Guo H Sun Q Liuli H Wang Y. Heat shock protein 19.9 (Hsp19.9) from *Bombyx mori* is involved in host protection against viral infection. Dev Comp Immunol. (2021) 114:103790. 10.1016/j.dci.2020.103790 32784012

[17] Guo HZ Xu GW Wang BB Xia F Sun Q Wang YM. Phosphoenolpyruvate carboxykinase is involved in antiviral immunity against *Bombyx mori* nucleopolyhedrovirus. Dev Comp Immunol. (2019) 92:193–98. 10.1016/j.dci.2018.11.015 30471302

[18] Jiang L Peng ZW Guo HZ Sun JC Sun Q Xia F. Enhancement of antiviral capacity of transgenic silkworms against cytoplasmic polyhedrosis virus via knockdown of multiple viral genes. Dev Comp Immunol. (2017) 77:138–40. 10.1016/j.dci.2017.07.020 28735962

[19] Zhao P Xia F Jiang L Guo H Xu G Sun Q. Enhanced antiviral immunity against *Bombyx mori* cytoplasmic polyhedrosis virus via overexpression of peptidoglycan recognition protein S2 in transgenic silkworms. Dev Comp Immunol. (2018) 87:84–9. 10.1016/j.dci.2018.05.021 29902708

[20] Sun Q Jiang L Guo H Xia F Wang B Wang Y. Increased antiviral capacity of transgenic silkworm via knockdown of multiple genes on *Bombyx mori* bidensovirus. Dev Comp Immunol. (2018) 87:188–92. 10.1016/j.dci.2018.06.002 29944898

[21] Liu W Liu J Lu Y Gong Y Zhu M Chen F. Immune signaling pathways activated in response to different pathogenic micro-organisms in *Bombyx mori*. Mol Immunol. (2015) 65:391–7. 10.1016/j.molimm.2015.02.018 25745806

[22] Hua X Li B Song L Hu C Li X Wang D. Stimulator of interferon genes (STING) provides insect antiviral immunity by promoting Dredd caspase-mediated NF-kappaB activation. J Biol Chem. (2018) 293:11878–90. 10.1074/jbc.RA117.000194 29875158 PMC6066306

[23] Swevers L Feng M Ren FF Sun JC. Antiviral defense against Cypovirus 1 (Reoviridae) infection in the silkworm, *Bombyx mori*. Arch Insect Biochem. (2020) 103:3. 10.1002/arch.21616 31502703

[24] Wang LL Cappelle K Santos D Vanden Broeck J Smagghe G Swevers L. Short-term persistence precedes pathogenic infection: infection kinetics of cricket paralysis virus in silkworm-derived Bm5 cells. J Insect Physiol. (2019) 115:1–1. 10.1016/j.jinsphys.2019.03.004 30905610

[25] Kingsolver MB Huang ZJ Hardy RW. Insect antiviral innate immunity: pathways, effectors, and connections. J Mol Biol. (2013) 425:4921–36. 10.1016/j.jmb.2013.10.006 24120681 PMC4007215

[26] Kim VN Han J Siomi MC Biogenesis of small RNAs in animals. Nature reviews. Molecular cell biology. (2009) 10:126–39. 10.1038/nrm2632 19165215

[27] Marques JT Imler JL. The diversity of insect antiviral immunity: insights from viruses. Curr Opin Microbiol. (2016) 32:71–6. 10.1016/j.mib.2016.05.002 27232381 PMC4983534

[28] Bronkhorst AW van Rij RP. The long and short of antiviral defense: small RNA-based immunity in insects. Curr Opin Virol. (2014) 7:19–28. 10.1016/j.coviro.2014.03.010 24732439

[29] Jayachandran B Hussain M Asgari S. RNA interference as a cellular defense mechanism against the DNA virus baculovirus. J Virol. (2012) 86:13729–734. 10.1128/JVI.02041-12 23055564 PMC3503065

[30] Zografidis A Van Nieuwerburgh F Kolliopoulou A Apostolou-Karampelis K Head SR Deforce D. Viral small-RNA analysis of *Bombyx mori* larval midgut during persistent and pathogenic cytoplasmic polyhedrosis virus infection. J Virol. (2015) 89:11473–86. 10.1128/JVI.01695-15 26339065 PMC4645660

[31] You L Zhang F Huang S Merchant A Zhou X Li Z. Over-expression of RNA interference (RNAi) core machinery improves susceptibility to RNAi in silkworm larvae. Insect Mol Biol. (2020) 29:353–62. 10.1111/imb.12639 32086963

[32] Leggewie M Schnettler E. RNAi-mediated antiviral immunity in insects and their possible application. Curr Opin Virol. (2018) 32:108–14. 10.1016/j.coviro.2018.10.004 30428425

[33] Zhu KY Palli SR. Mechanisms, applications, and challenges of insect RNA interference. Ann Rev Entomol. (2020) 65:293–311. 10.1146/annurev-ento-011019-025224 31610134 PMC9939233

[34] Asgari S MicroRNA functions in insects. Insect Biochem Mol Biol. (2013) 43:388–97. 10.1016/j.ibmb.2012.10.005 23103375

[35] Singh CP Singh J Nagaraju J. A baculovirus-encoded MicroRNA (miRNA) suppresses its host miRNA biogenesis by regulating the exportin-5 cofactor Ran. J Virol. (2012) 86:7867–9. 10.1128/JVI.00064-12 22593162 PMC3421685

[36] Singh CP Singh J Nagaraju J. bmnpv-miR-3 facilitates BmNPV infection by modulating the expression of viral P6.9 and other late genes in *Bombyx mori. Insect Biochem Mol Biol*. (2014) 49:59–69. 10.1016/j.ibmb.2014.03.008 24698834

[37] Guo JY Wang YS Chen T Jiang XX Wu P Geng T. Functional analysis of a miRNA-like small RNA derived from *Bombyx mori* cytoplasmic polyhedrosis virus. Insect science. (2020) 27:449–62. 10.1111/1744-7917.12671 30869181

[38] Wu P Jiang X Sang Q Annan E Cheng T Guo X. Inhibition of miR-274-3p increases BmCPV replication by regulating the expression of BmCPV NS5 gene in *Bombyx mori*. Virus Genes. (2017) 53:643–9. 10.1007/s11262-017-1466-7 28493152

[39] Wu P Shang Q Dweteh OA Huang H Zhang S Zhong J. Over expression of bmo-miR-2819 suppresses BmNPV replication by regulating the BmNPV ie-1 gene in *Bombyx mori*. Mol Immunol. (2019) 109:134–9. 10.1016/j.molimm.2019.03.013 30947109

[40] Wu P Qin G Qian H Chen T Guo X. Roles of miR-278-3p in IBP2 regulation and *Bombyx mori* cytoplasmic polyhedrosis virus replication. Gene. (2016) 575(2 Pt 1):264–9. 10.1016/j.gene.2015.09.009 26348138

[41] Kolliopoulou A Santos D Taning CNT Wynant N Vanden Broeck J Smagghe G PIWI pathway against viruses in insects. Wires Rna. (2019) 10:6 10.1002/wrna.1555 31183996

[42] Feng M Kolliopoulou A Zhou YH Fei SG Xia JM Swevers L. The piRNA response to BmNPV infection in the silkworm fat body and midgut. Insect Sci. (2020) 10.1111/1744-7917.12796 32367653

[43] Katsuma S Kawamoto M Shoji K Aizawa T Kiuchi T Izumi N. Transcriptome profiling reveals infection strategy of an insect maculavirus. DNA Res. (2018) 25:277–86. 10.1093/dnares/dsx056 29360973 PMC6014269

[44] Hoffmann JA The immune response of Drosophila. Nature. (2003) 426:33–8. 10.1038/nature02021 14603309

[45] Kaneko T Yano T Aggarwal K Lim JH Ueda K Oshima Y. PGRP-LC and PGRP-LE have essential yet distinct functions in the drosophila immune response to monomeric DAP-type peptidoglycan. Nat Immunol. (2006) 7:715–23. 10.1038/ni1356 16767093

[46] Mussabekova A Daeffler L Imler JL. Innate and intrinsic antiviral immunity in Drosophila. Cell Mol Life Sci. (2017) 74:2039–54. 10.1007/s00018-017-2453-9 28102430 PMC5419870

[47] Zambon RA Nandakumar M Vakharia VN Wu LP. The Toll pathway is important for an antiviral response in Drosophila. Proc Natl Acad Sci USA. (2005) 102:7257–62. 10.1073/pnas.0409181102 15878994 PMC1129099

[48] Lamiable O Kellenberger C Kemp C Troxler L Pelte N Boutros M. Cytokine Diedel and a viral homologue suppress the IMD pathway in Drosophila. Proc Natl Acad Sci USA. (2016) 113:698–703. 10.1073/pnas.1516122113 26739560 PMC4725508

[49] Feng M Fei SG Xia JM Labropoulou V Swevers L Sun JC. Antimicrobial peptides as potential antiviral factors in insect antiviral immune response. Front Immunol. (2020) 11. 10.3389/fimmu.2020.02030 32983149 PMC7492552

[50] Hombria JC Brown S. The fertile field of Drosophila Jak/STAT signalling. Curr Biol. (2002) 12:R569–75. 10.1016/S0960-9822(02)01057-6 12194841

[51] Paradkar PN Trinidad L Voysey R Duchemin JB Walker PJ. Secreted Vago restricts West Nile virus infection in Culex mosquito cells by activating the Jak-STAT pathway. Proc Natl Acad Sci USA. (2012) 109:18915–20. 10.1073/pnas.1205231109 23027947 PMC3503207

[52] Souza-Neto JA Sim S Dimopoulos G. An evolutionary conserved function of the JAK-STAT pathway in anti-dengue defense. Proc Natl Acad Sci USA. (2009) 106:17841–6. 10.1073/pnas.0905006106 19805194 PMC2764916

[53] Dostert C Jouanguy E Irving P Troxler L Galiana-Arnoux D Hetru C. The Jak-STAT signaling pathway is required but not sufficient for the antiviral response of drosophila. Nat Immunol. (2005) 6:946–53. 10.1038/ni1237 16086017

[54] Hu X Zhang X Wang J Huang M Xue R Cao G. Transcriptome analysis of BmN cells following over-expression of BmSTAT. Mol Gen Gen. (2015) 290:2137–46. 10.1007/s00438-015-1065-z 25998838

[55] Zhang X Guo R Kumar D Ma H Liu J Hu X. Identification, gene expression and immune function of the novel Bm-STAT gene in virus-infected *Bombyx mori*. Gene. (2016) 577:82–8. 10.1016/j.gene.2015.11.027 26592694

[56] Shang Q Wu P Huang HL Zhang SL Tang XD Guo XJ. Inhibition of heat shock protein 90 suppresses *Bombyx mori* nucleopolyhedrovirus replication in *B. mori*. Insect Mol Biol. (2020) 29:205–13. 10.1111/imb.12625 31621968

[57] Toufeeq S Wang J Zhang SZ Li B Hu P Zhu LB. Bmserpin2 is involved in BmNPV infection by suppressing melanization in *Bombyx mori*. Insects. (2019) 10(11). 10.3390/insects10110399 31717928 PMC6921080

[58] Jiang L Liu WQ Guo HZ Dang YH Cheng TC Yang WY. Distinct functions of *Bombyx mori* peptidoglycan recognition protein 2 in immune responses to bacteria and viruses. Front Immunol. (2019) 10:776. 10.3389/fimmu.2019.00776 31031766 PMC6473039

[59] Katsuma S Mita K Shimada T. ERK- and JNK-Dependent signaling pathways contribute to *Bombyx mori* nucleopolyhedrovirus infection. J Virol. (2007) 81:13700–9. 10.1128/JVI.01683-07 17913811 PMC2168829

[60] Guo HZ Sun Q Wang BB Wang YM Xie EY Xia QY. Spry is downregulated by multiple viruses to elevate ERK signaling and ensure viral reproduction in silkworm. Dev Comp Immunol. (2019) 98:1–5. 10.1016/j.dci.2019.04.001 30965060

[61] Gonzalez-Santoyo I Cordoba-Aguilar A Phenoloxidase: a key component of the insect immune system. Entomol Exp Appl. (2012) 142:1–16. 10.1111/j.1570-7458.2011.01187.x

[62] Cerenius L Lee BL Soderhall K. The proPO-system: pros and cons for its role in invertebrate immunity. Trends Immunol. (2008) 29:263–71. 10.1016/j.it.2008.02.009 18457993

[63] Dudzic JP Hanson MA Iatsenko I Kondo S Lemaitre B. More than black or white: melanization and toll share regulatory serine proteases in Drosophila. Cell Rep. (2019) 27:1050. 10.1016/j.celrep.2019.03.101 31018123

[64] Yuan CF Xing LS Wang ML Wang X Yin MY Wang QR. Inhibition of melanization by serpin-5 and serpin-9 promotes baculovirus infection in cotton bollworm Helicoverpa armigera. PLoS Pathog. (2017) 13:6645. 10.1371/journal.ppat.1006645 28953952 PMC5633200

[65] Wang QR Yin MY Yuan CF Liu XJ Hu ZH Zou Z. Identification of a conserved prophenoloxidase activation pathway in cotton bollworm *Helicoverpa armigera*. Front Immunol. (2020) 11:785. 10.3389/fimmu.2020.00785 32431706 PMC7215089

[66] Xiao W Yang Y Weng QB Lin TH Yuan MJ Yang K. The role of the PI3K-Akt signal transduction pathway in *Autographa californica* multiple nucleopolyhedrovirus infection of *Spodoptera frugiperda* cells. Virology. (2009) 391:83–89. 10.1016/j.virol.2009.06.007 19573890

[67] Datta SR Brunet A Greenberg ME. Cellular survival: a play in three Akts. Gene Dev. (1999) 13:2905–27. 10.1101/gad.13.22.2905 10579998

[68] Fruman DA Chiu H Hopkins BD Bagrodia S Cantley LC Abraham RT The PI3K pathway in human disease. Cell. (2017) 170:605–35. 10.1016/j.cell.2017.07.029 28802037 PMC5726441

[69] Vanhaesebroeck B Guillermet-Guibert J Graupera M Bilanges B. The emerging mechanisms of isoform-specific PI3K signalling. Nat Rev Mol Cell Bio. (2010) 11:329–41. 10.1038/nrm2882 20379207

[70] Engelman JA. Targeting PI3K signalling in cancer: opportunities, challenges and limitations. Nat Rev Cancer. (2009) 9:550–62. 10.1038/nrc2664 19629070

[71] Jiramongkol Y Lam EWF. FOXO transcription factor family in cancer and metastasis. Cancer Metast Rev. (2020) 39:681–709. 10.1007/s10555-020-09883-w 32372224 PMC7497309

[72] Kang X Wang Y Liang W Tang X Zhang Y Wang L. *Bombyx mori* nucleopolyhedrovirus downregulates transcription factor BmFoxO to elevate virus infection. Dev Comp Immunol. (2020) 10:3904. 10.1016/j.dci.2020.103904 33245980

[73] Lee YR Chen M Pandolfi PP. The functions and regulation of the PTEN tumour suppressor: new modes and prospects. Nat Rev Mol Cell Bio. (2018) 19:547–62. 10.1038/s41580-018-0015-0 29858604

[74] Diehl N Schaal H. Make yourself at home: viral hijacking of the PI3K/Akt signaling pathway. Viruses-Basel. (2013) 5:3192–212. 10.3390/v5123192 24351799 PMC3967167

[75] Cooray S. The pivotal role of phosphatidylinositol 3-kinase-Akt signal transduction in virus survival. J Gen Virol. (2004) 85:1065–76. 10.1099/vir.0.19771-0 15105524

[76] Wang B Jiang L Guo H Sun Q Wang Y Xie E. Screening of PI3K-Akt-targeting drugs for silkworm against *Bombyx mori* nucleopolyhedrovirus. Molecules. (2019) 24:7. 10.3390/molecules24071260 30939726 PMC6480691

[77] Shaul YD Seger R. The MEK/ERK cascade: from signaling specificity to diverse functions. Biochimica et biophysica acta. (2007) 1773:1213–26. 10.1016/j.bbamcr.2006.10.005 17112607

[78] Hayashi S Ogura Y. ERK signaling dynamics in the morphogenesis and homeostasis of Drosophila. Curr Opin Genet Dev. (2020) 63:9–15. 10.1016/j.gde.2020.01.004 32145545

[79] Johnson GL Lapadat R. Mitogen-activated protein kinase pathways mediated by ERK, JNK, and p38 protein kinases. Science. (2002) 298:1911–2. 10.1126/science.1072682 12471242

[80] Bonjardim CA. Viral exploitation of the MEK/ERK pathway - A tale of vaccinia virus and other viruses. Virology. (2017) 507:267–75. 10.1016/j.virol.2016.12.011 28526201

[81] Morrison DK MAP kinase pathways. Csh Perspect Biol. (2012) 4(11). 10.1101/cshperspect.a011254 PMC3536342 23125017

[82] Buday L Downward J. Epidermal growth-factor regulates P21(Ras) through the formation of a complex of receptor, Grb2 adapter protein, and sos nucleotide exchange factor. Cell. (1993) 73:611–20. 10.1016/0092-8674(93)90146-H 8490966

[83] Jin S Cheng T Guo Y Lin P Zhao P Liu C. *Bombyx mori* epidermal growth factor receptor is required for nucleopolyhedrovirus replication. Insect Mol Biol. (2018) 27:464–77. 10.1111/imb.12386 29603500

[84] Jin SK Cheng TC Jiang L Lin P Yang Q Xiao Y. Identification of a new sprouty protein responsible for the inhibition of the *Bombyx mori* Nucleopolyhedrovirus reproduction. PLoS ONE. (2014) 9:e99200. 10.1371/journal.pone.0099200 24915434 PMC4051654

[85] Levitzki A Gazit A. Tyrosine Kinase Inhibition - an Approach to Drug Development. Science. (1995) 267:1782–8. 10.1126/science.7892601 7892601

[86] Favata MF Horiuchi KY Manos EJ Daulerio AJ Stradley DA Feeser WS. Identification of a novel inhibitor of mitogen-activated protein kinase kinase. J Biol Chem. (1998) 273:18623–32. 10.1074/jbc.273.29.18623 9660836

[87] Jiang L Goldsmith MR Xia QY Advances in the arms race between silkworm and baculovirus. Front Immunol. (2021) 12:628151 10.3389/fimmu.2021.628151 PMC7900435 33633750

